# A computed tomography study investigating the effects of botulinum toxin injections prior to complex abdominal wall reconstruction

**DOI:** 10.1007/s10029-022-02692-w

**Published:** 2022-10-14

**Authors:** J. J. M. Claessen, A. S. Timmer, R. Hemke, J. J. Atema, R. Hompes, M. A. Boermeester, M. V. H. Rutten

**Affiliations:** 1 Department of Surgery (Suite J1A-228), Amsterdam UMC, University of Amsterdam, Location AMC, Meibergdreef 9, 1105AZ Amsterdam, The Netherlands; 2 Department of Radiology, Amsterdam UMC, University of Amsterdam, Amsterdam, The Netherlands; 3 Department of Anesthesiology, Amsterdam UMC, University of Amsterdam, Amsterdam, The Netherlands; 4 Amsterdam Gastroenterology and Metabolism, Amsterdam, The Netherlands

**Keywords:** Botulinum toxin A, CAWR, CT scan

## Abstract

**Objective:**

To explore how intramuscular injection of botulinum toxin A (BTA) affects the lateral abdominal wall (LAW) musculature, abdominal- and hernia dimensions, and muscle structure on computed tomography (CT) in patients scheduled for complex abdominal wall reconstruction (CAWR).

**Methods:**

Retrospective analysis of prospectively registered patients who received bilateral intramuscular BTA injections into all three muscles of the LAW. Only patients for which a CT was available before and 3–6 weeks after BTA treatment prior to surgery were analyzed.

**Results:**

Fifty-two patients were analyzed. Median hernia width in all patients decreased with 0.4 cm (IQR − 2.1;0.6) (*p* = 0.023). Median intra-abdominal transverse diameter increased with 0.9 cm (IQR − 0.2;3.3) (*p* = 0.001) and the intra-abdominal anterior–posterior diameter decreased with 0.5 cm (IQR − 1.3;0.5) (*p* = 0.017), making the abdomen more oval. Median LAW muscle length increased with 0.9 cm (IQR 0.0;2.4) per side (*p* < 0.001), muscle thickness decreased with 0.5 cm (IQR − 0.8;− 0.2) (− 25.0%) per side (*p* < 0.001), and muscle mass decreased with 3.9 cm^2^ (IQR − 6.4;-1.5) (− 15.8%) per side (*p* < 0.001). Median HU of the psoas muscles (density) increased with 4.8 HU (IQR 0.4;9.7) (10.3%) per side (*p* < 0.001). Effects of BTA were more pronounced in patients with a loss of domain (LoD) ≥ 20%.

**Conclusions:**

The main effect of BTA injections is elongation and thinning of the LAW muscles, more than a decrease in hernia width. Concomitantly, the abdomen becomes more oval. An increase of psoas muscles density is seen, associated with offloading of the LAW muscles. Patients with large LoD have a proportionally higher effect of BTA.

**Supplementary Information:**

The online version contains supplementary material available at 10.1007/s10029-022-02692-w.

## Introduction

Repair of complex incisional hernia poses a great challenge. The goal of such repair is to restore the original anatomy by a tension-free (mesh-reinforced) fascial closure. This is often not possible in complex cases, such as massive hernias, chronically retracted lateral abdominal wall (LAW) muscles, and/or large loss of domain (LoD) [1]. Chronic muscle retraction usually reduces the volume of the abdominal cavity and enlarges the size and volume of the hernia. This hampers reduction of hernia sac content into the abdominal cavity and fascial closure during abdominal wall reconstruction (AWR). As such, patients whose formal fascial layers are not closed during the repair and undergo the so-called bridging mesh repair are at higher risk to develop a hernia recurrence [2, 3]. Reduction of a large hernia sac content in the abdominal cavity in cases of LoD increases the risk of abdominal compartment syndrome or ventilatory problems postoperatively [4, 5].

Surgical myofascial reconstruction techniques, also known as component separation techniques (CST), can facilitate fascial closure during complex AWR (CAWR), but are associated with an increased risk of surgical site infection, hematoma, and seroma formation [3, 7]. Other techniques, such as progressive pneumoperitoneum (PPP) and soft-tissue expanders, are associated with other types of risks [7, 8].

Botulinum toxin type A (BTA) is a neurotoxin derived from the bacteria *Clostridium botulinum*. When injected into muscle tissue, it causes a temporary muscle paralysis [9]. Its paralyzing effect maximizes around two to four weeks after injection and decreases in the 2–4 months after [10]. The injection of BTA in the LAW muscles prior to AWR was first described in 2009 [11]. Since then, neoadjuvant use of BTA has acquired general acceptance in AWR. Recent studies propose that abdominal wall compliance is increased which facilitates fascial medialization, and possibly even precludes the need to perform surgical CST [12–14]. Further advantages of BTA are lack of adverse effects and the temporary, instead of permanent, working mechanism. Moreover, the muscle relaxation continues in the first few months after surgery giving the abdominal cavity and its contents time to adapt to the reconstructed situation and to protect fascial healing [15, 16]. Despite positive preliminary results, much is still unknown about the true effects of BTA as well as the extent thereof in high-risk patients with complex abdominal wall defects after multiple abdominal operations and reconstructions. Only a few studies have reported pre- and post injection measurements of the LAW musculature and transverse hernia width [16–21]. Furthermore, the effects of BTA on LoD and body composition measures have not yet been investigated.

The value of computed tomography (CT) derived body composition measures for predicting postoperative outcomes has evolved the last couple of years in numerous surgical specialties [22–25]. It has been suggested that measurements on CT may provide objective and independent measures of muscle structure [26]. Lower muscle mass is an indication of muscle atrophy. Lower muscle density, also known as lower muscle attenuation, results from an accumulation of intramuscular lipid depositions (myosteatosis), and the presence thereof on CT can be shown through a decrease in muscle density [27–29]. Low muscle mass and low muscle density both lead to a decrease in muscle structure and therefore in muscle strength [30].

The aim of this explorative study is to assess how intramuscular injection of BTA affects the LAW musculature, abdominal- and hernia dimensions, and muscle structure on CT in patients scheduled for CAWR.

## Methods

### Study design and patient selection

In this observational study, a retrospective analysis was performed of consecutive prospectively registered patients scheduled for CAWR and pretreated with BTA in the Amsterdam UMC, location AMC, The Netherlands. Patients were selected for BTA injections if they had large defects (> 10 cm), and/or large LoD (≥ 20%). Patients with abdominal wall defects < 10 cm with or without complicating factors such as intestinal fistula and infected mesh received BTA to achieve fascial closure without the need for a component separation technique*.* For this study, we selected patients that received bilateral BTA injections into all three muscles of the LAW. Only patients in whom a CT was performed before and 3 to 6 weeks after BTA treatment were included. Patients that received unilateral treatment, or selective injection in only one or two muscle layers were excluded. All patients gave consent for use of their data. The study is reported following the Strengthening the Reporting of Observational Studies in Epidemiology (STROBE) statement [31].

### Injection technique

Our standard protocol consisted of six BTA injections - three at both sides - located between the lower costal border and the anterior superior iliac spine, on the anterior axillary line. Deviations occurred due to patient history, comorbidities such as skin defects and wounds, or distorted anatomy. Using ultrasound guidance, BTA was deposited in the transversus abdominis muscle after which the needle was retracted into the internal oblique muscle in which a similar amount of BTA was deposited followed by retracting the needle once more into the external oblique muscle for the last BTA deposit. Six hundred units of abobotulinumtoxin A (Dysport^®^) were diluted in 120 ml of 0.9% saline. This was distributed into six separate 20 ml syringes (5 units BTA per ml). For every injection, a single syringe was used. At each of the six locations, 6–8 ml of Dysport 5 units/ml was injected into each separate muscle of the LAW. All BTA injections were performed by 1 of 2 dedicated BTA anesthesiologists.

### Data items

Baseline demographic data include age, sex, body mass index (BMI), location of the hernia (midline or lateral), and number of previous abdominal surgeries. Abdominal wall defects were classified according to the modified Ventral Hernia Working Group grading system (mVHWG) [32]. We further assigned them to one of three severity classes (minor, moderate, and major complex) as described in the Slater complexity classification by an expert consensus group in 2014 [33]. To encompass all hernia characteristics, we registered several important hernia- and wound-specific risk factors present, including presence of stomata, presence of intestinal fistula(s), presence of infected mesh, transverse defect width ≥ 10 cm, LoD ≥ 20%, and previous hernia repair [34].

**Fig. 1:**
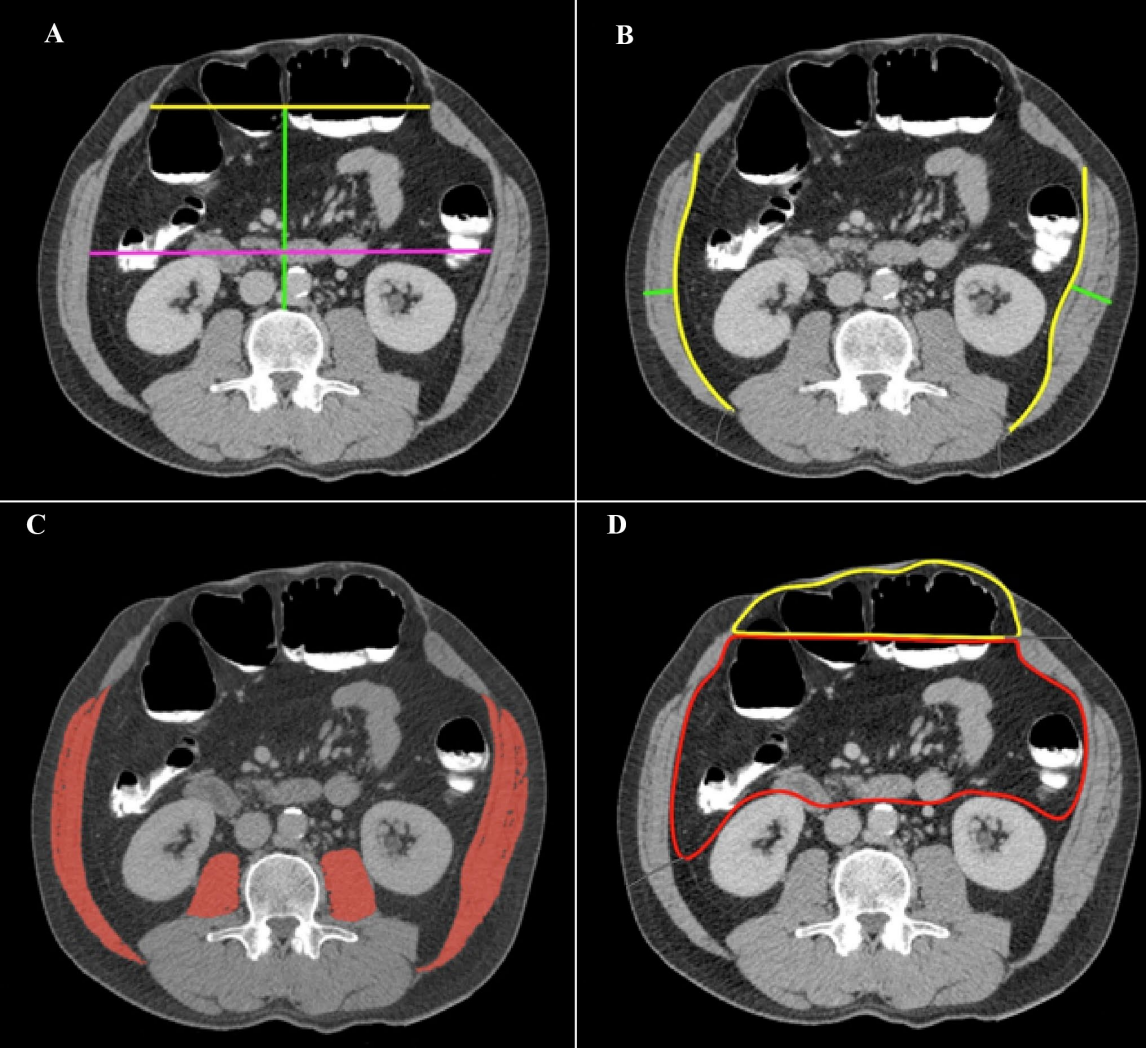
Measurements. **A** hernia width (yellow line), intra-abdominal width (purple line), and intra-abdominal depth (green line); **B** LAW muscle length (yellow line) and LAW muscle thickness (green line); **C** LAW muscle mass and density, and psoas muscle mass and density; **D** The intra-abdominal volumes and hernia volumes were obtained by encircling the intra-abdominal (red) and hernia (yellow) surfaces on all axial CT slides from the diaphragm to the pelvis. Then, the segmentation tool calculated the volumes. Loss of domain was calculated according to the Sabbagh method (hernia volume/(hernia- + abdominal volume)). *LAW* lateral abdominal wall

CT measurements were performed using a DICOM (digital imaging and communications in medicine) viewer, open-source software (HOROS, www.horosproject.org). Figure 1 illustrates how all measurements were performed. Lateral abdominal wall muscle length measurements were taken along the inner surface of the transversus abdominis muscle, from the dorsolateral edge of quadratus lumborum muscle to the lateral edge of rectus abdominis muscle. Muscle thickness of the LAW was measured from the lateral edge of the external oblique muscle to the medial edge of transversus abdominis, halfway of the LAW muscles and perpendicular to the direction of the muscle fibers (Fig. 1a). Measurements of the LAW muscle length, LAW muscle thickness, LAW muscle mass, and muscle attenuation (density) were obtained on a single axial CT-slide at the third vertebral level (L3) on both the pre- and post BTA scan. All measurements of the LAW were taken on both the right and left side separately. Measurements of the transverse hernia diameter (hernia width), intra-abdominal transverse diameter (abdominal width), and intra-abdominal anterior–posterior diameter (abdominal depth) were obtained on the pre-BTA scan on a single axial CT-slide at the axial level at which these measurements were the largest (Fig. 1b). For each patient individually, the post BTA measurements were obtained at the exact same level. The level at which the abdominal width and -depth was measured was always located between the lower costal border and the upper limit of the iliac crest. For muscle mass, the muscle cross-sectional surface (cm^2^) of the LAW muscles was identified and quantified by HU thresholds of − 30 to + 150, for each side separately (Fig. 1c) [25, 35]. The muscle density of the LAW and psoas muscles was determined by calculating the average HU of the muscles surface [29, 36]. Since contrast administration has an effect on muscle density, these measurements were exclusively performed on CT scans that were performed using intravenous contrast administration. Hernia width, abdominal width, and abdominal depth were only measured in patients with a midline defect. When it was not possible to obtain measurements form both sides, e.g., due to a lateral defect, destroyed muscle fibers, or incomplete visualization of the muscle, only the intact side was measured.

Measurements of the intra-abdominal- and hernia volume were obtained using a segmentation tool. The boundaries of the specified volumes were manually encircled - on all axial CT slides from the diaphragm to the pelvis - after which the segmentation tool calculated the volume (Fig. 1d). The abdominal volume consisted of the true intra-abdominal content, excluding the retroperitoneal space. A line between the medial edges of the recti muscles was drawn to separate the abdominal volume from the hernia volume. LoD was expressed as the hernia volume/total peritoneal volume (hernia + abdominal volume) according to the method of Sabbagh et al [37].

Surgical data encompassed use of a component separation technique [CST; anterior CST, transverse abdominis release (TAR)], type of mesh repair, mesh location, and posterior and/or anterior fascial closure or bridged repair. Preparation over the posterior rectus sheath with mesh placement in Rives-Stoppa was not noted as a CST*.* For mesh strategy and surgical techniques, see Supplement S1.

### Analysis

Numerical data are expressed as medians and interquartile ranges (IQR). Pre- and post BTA data were compared using the Wilcoxon signed-rank test, with a significant level *p* < 0.05. Stratification was performed according to LoD ≥ 20%, number of abdominal surgeries ≥ 3, and number of previous repairs ≥ 1. In addition, results of patients with a defect of < 10 cm were compared to the results of patients with a defect of ≥ 10 cm. Furthermore, a comparison was made between patients with- and without persistent inflammation or contamination at the time of BTA treatment [i.e., patients with enterocutaneous or -atmospheric fistula (ECF/EAF), infected mesh, or intra-abdominal abscess].

## Results

### Participant selection

Between October 2018 and July 2021, 62 patients received BTA injections prior to CAWR. Of these, 9 patients did not have a pre- or post BTA CT, and 1 patient did not receive BTA in all three muscle layers. Therefore, a total of 52 patients were included in this study.

### Baseline characteristics

Baseline patient- and hernia characteristics of all included patients are summarized in Table 1. Median BMI was 29.2 kg/m^2^ (IQR 25.7;32.3) and over 40% of patients was obese (BMI > 30 kg/m^2^). Forty-five patients presented with a midline defect and seven with a lateral defect. Forty-five patients (86.5%) had one or more complicating hernia characteristics. Forty-one patients (78.9%) had at least 3 previous abdominal surgeries with over half of all patients (51.9%) had undergone one or more previous hernia repairs. Pre-BTA median hernia width was 14.0 cm (IQR 8.9;18.6) and pre-BTA median LoD was 10% (IQR 3.0;19.2). Time between BTA treatment and surgery was 39 days (IQR 30;44) and between the pre- and post BTA CT was 259 days (IQR 125;367).

**Table 1:** Patient- and hernia characteristics

	Total group (*n* = 52)
Age (years), median (IQR)	56 (46.3;65.8)
Male sex, no (%)	28 (53.8%)
BMI (kg/m^2^), median (IQR)	29.2 (25.7;32.3)
BMI > 30 (kg/m^2^), no (%)	21 (40.3%)
Location of hernia, no (%)	
Midline Lateral	45 (86.5% 7 (13.5%)
Complicating hernia characteristics (ǂ), no (%)	
0 1–2 ≥ 3	7 (13.5%) 30 (57.7%) 15 (28.8%)
Modified VHWG classification, grade, no (%)	
1 2 3	5 (9.6%) 23 (44.2%) 24 (46.2%)
Hernia complexity according to Slater classification, no (%)	
Minor complex Moderate complex Major complex	2 (3.8%) 23 (44.2%) 27 (52%)
Hernia width (cm), median (IQR)	14 (8.9;18.6)
Loss of domain (%), median (IQR)	10 (3.0;19.2)
Stoma present, any kind, no (%)	10 (19.2%)
Intestinal fistula(s) present, no (%)	12 (23.1%)
Infected mesh present, no (%)	9 (17.3%)
Previous abdominal surgery, no (%)	
1–2 3–4 ≥ 5	11 (21.1%) 21 (40.4%) 20 (38.5%)
Previous hernia repair, no (%)	
0 1 ≥ 2	25 (48.1%) 13 (25%) 14 (26.9%)

(ǂ) Including: presence of a stoma, intestinal fistula, infected mesh, transverse defect width ≥ 10 cm, loss of domain > 20%, and previous hernia repair)

*BMI* body mass index, *VHWG* ventral hernia working grade

### Muscle-, abdominal-, and hernia dimensions

**Fig. 2:**
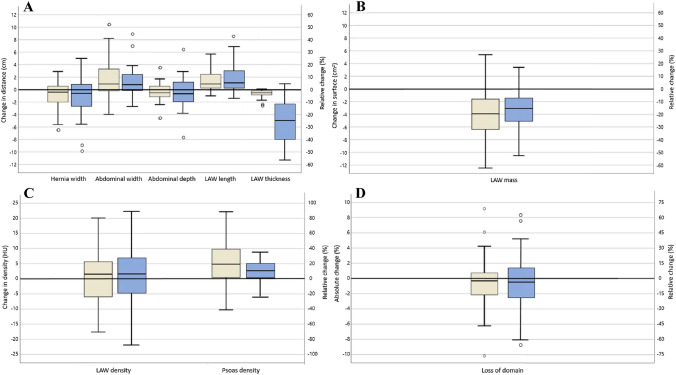
Box plots of the differences between median pre- and post-injection measures (gold boxplots) and individual relative changes compared to pre-BTA (blue boxplots): **A** hernia width, abdominal width and -depth, and LAW length and -thickness, **B** LAW muscle mass, **C** LAW muscle density and psoas muscle density, and **D** loss of domain. The boxes indicate median and interquartile range. The transverse line is de zero-line. *LAW* lateral abdominal wall, *LoD* loss of domain, *HU* Hounsfield units

**Fig. 3:**
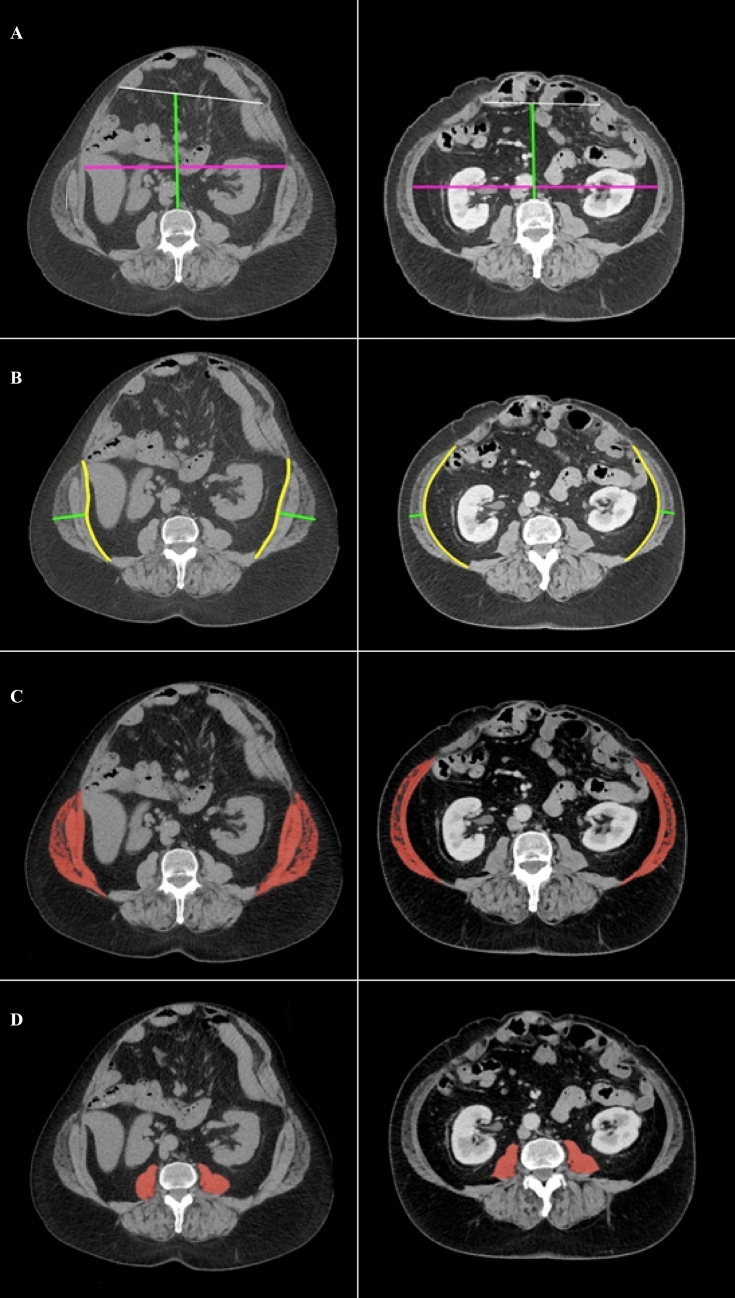
An example of showing optimal effects of pre- and post-BTA treatment, although such effects were not observed in all patients. **A** Hernia width (white line) and abdominal width (purple line) and abdominal depth (green line), **B** LAW muscle length (yellow line) and LAW muscle thickness (green line), and **C** LAW muscle mass and LAW muscle density. **D** Psoas muscle density. *LAW* lateral abdominal wall

The differences between the pre- and post BTA measurements and relative changes for individual patients are shown in boxplots in Fig. 2. An example of optimal BTA effects on muscle measurements and hernia- and abdominal dimensions is shown in Fig. 3.

Results for the overall group, and by stratification on LoD, number of abdominal surgeries, and number of previous repairs are depicted in Supplement S2. Results of a direct comparison between patients with a defect of < 10 cm and patients with a defect of ≥ 10 cm are shown in Supplement S3. A comparison between patients with and without persistent inflammation or contamination is shown in Supplement S5.

#### Hernia width

Median hernia width decreased with 0.4 cm (IQR − 2.1;0.6) (*p* = 0.023), resulting in a 2.9% reduction. As shown by the boxplot, BTA has a heterogeneous effect on hernia width, as nearly half of all patients demonstrated an increase.

In patients with pre-BTA LoD ≥ 20%, median decrease in hernia width after BTA was 2.7 cm (IQR − 5.5;0.7) (*p* = 0.051), 14.0% decrease (Supplement S2). In patients with a pre-BTA hernia width of ≥ 10 cm, a median decrease of 1.0 cm (IQR − 2.4;0.6; *p* = 0.014) was observed after BTA, whereas a significant decrease was not seen for those with a pre-BTA hernia width of less than 10 cm (0.1 cm (IQR − 0.4;0.5) (Supplement S3).

In Supplement S4, we compared patient-, hernia-, and surgical characteristics between patients with decreased hernia width and patients with an increased hernia width after BTA for possible differences. All these variables were comparable among BTA hernia width responding and non-responding groups.

#### Abdominal width and -depth

Abdominal width increased with 0.9 cm (IQR − 0.2;3.3) (*p* = 0.001) (3.3% increase), whereas abdominal depth decreased with 0.5 cm (IQR − 1.3;0.5) (*p* = 0.045) (3.5% reduction). The abdomen adopted a more oval shape. In approximately 30% of all patients, the opposite occurred as shown by the boxplots, being a decrease in abdominal width and an increase in abdominal depth. This explains why the overall median change in abdominal width and -depth is relatively small, whereas individual changes may be larger. In patients with a pre-BTA LoD of at least 20%, abdominal width increased with 2.9 cm (IQR 0.4;3.8) (*p* = 0.022), being an 11.1% increase (Supplement S2).

#### LAW muscle length

Median length of the LAW increased 0.9 cm per side (IQR 0.0;2.4) (*p* < 0.001) (5.4% increase per side). About 20% of all patients had a decrease in length instead of an increase. In patients with pre-BTA LoD ≥ 20%, median increase per side was 1.7 cm (IQR 0.6;2.6) (*p* < 0.001), 10.4% increase per side (Supplement S2). Patients with a pre-BTA hernia width of ≥ 10 cm showed a median increase of LAW muscle length of 1.0 cm per side (IQR 0.0;2.3) (*p* < 0.001) after BTA, comparable to those with a pre-BTA width of less than 10 cm (0.9 (IQR 0.0;3.3), *p* < 0.007; *p* = 0.922) (Supplement S3).

#### LAW muscle thickness

Median thickness of the LAW muscles decreased with 0.5 cm per side (IQR − 0.8;− 0.2) (*p* < 0.001); a reduction of 25% per side. Consistently in nearly all patients, a decrease in thickness of the LAW muscles was found. In patients with a LoD ≥ 20%, a decrease of 0.8 cm (IQR − 1.7;− 0.5) in LAW muscle thickness per side was observed (*p* < 0.001), being a reduction of 34.8% per side (Supplement 2). For patients having a pre-BTA hernia width of more than 10 cm, a decrease in LAW muscle thickness of 0.6 cm per side was found after BTA (IQR − 0.8;− 0.2) (*p* < 0.001), comparable to those with a hernia < 10 cm (− 0.5 (IQR − 0.9;0.2; *p* < 0.001) (Supplement 3).

#### LAW muscle mass and -density

Median LAW muscle mass decreased with 3.9 cm^2^ (IQR − 6.4;− 1.5) per side (*p* < 0.001); a 15.8% reduction. However, about 20% of all patients experienced an increase in muscle mass of the LAW. A decrease of 6.8 cm^2^ (IQR − 10.9;− 3.5), (*p* = 0.001) per side was found in patients with LoD ≥ 20%; a reduction of 30.3% per side. No significant effect on LAW muscle density was seen, both for the study population and for stratified subgroups.

#### Loss of domain

Overall, no significant effect on LoD was observed. However, in patients with a pre-BTA LoD ≥ 20%, a significant decrease in LoD was observed with an absolute reduction of 3.7% (IQR − 12.0;− 2.1) (*p* = 0.012) and a relative decrease of 13.8% (Supplement S2). For patients with a pre-BTA hernia width of ≥ 10 cm, a small median absolute reduction of 0.9% (IQR − 2.7;1.2; *p* = 0.140) was observed after BTA, whereas this decrease was median 0.0% (IQR − 1.2;0.2; *p* = 0.388) for those with a pre-BTA hernia width < 10 cm (Supplement S3).

#### Psoas muscle density

Median HU of the psoas muscles increased with 4.8 HU (IQR 0.4;9.7) per side (*p* < 0.001), an increase of 10.3%. However, in about 25% of patients, a decrease in muscle density of the psoas muscles was seen.

#### Subgroups according to loss of domain, previous abdominal surgery, previous hernia repair, or hernia width

Overall, BTA in patients with three or more previous abdominal operations or at least one previous hernia repair was comparably effective as shown for the entire study population. BTA effects in patients with LoD of at least 20% were more pronounced compared to the data of entire cohort (Supplement S2). Furthermore, BTA seems to be more effective by and large in patients with a defect of  ≥ 10 cm, although statistically no significant differences were found among measured variables when compared to patients with a defect of < 10 cm (Supplement S3)*.*

#### Subgroup according to persistent inflammation or contamination

In the group of patients without persistent inflammation or contamination at the time of BTA, median increase of abdominal width [1.5 cm (IQR − 0.2;3.4), *p* = 0.001 vs pre-BTA] was significantly higher than in the group with persistent inflammation or contamination (*p* = 0.035 among groups). All other measured dimensions were comparable among the two groups, but BTA effects were, in general, non-significantly smaller in patients with persistent inflammation or contamination (Supplement S5).

### Surgical data

Surgical data are depicted in Table 2. Overall, a CST was performed in 28 of 52 patients (58.8%) with anterior lateral release or (less often) open ACS in 16 (30.7%) and TAR in 11 (21.2%) patients. A single mesh was used in 75% (39 of 52 patients) and a double-layer technique in 25% (13 of 52 patients). Complete fascial closure was achieved in 38 of 52 patients (73.1%), whereas a bridged repair was performed in 8 patients (15.4%). Closure of only the anterior fascia occurred in 2 of 52 patients (3.8%) and closure of just posterior fascia in 4 patients (7.7%). In patients with a defect size < 10 cm, a CST was performed in 31.3% (5 of 16 patients). In patients with a defect size ≥ 10 cm, a CST was performed in 63.9% (23 of 36 patients). Complete fascial closure was obtained in 13 of 16 (81.1%) patients with a defect size < 10 cm. When patients had a defect size of ≥ 10 cm, complete fascial closure was achieved in 25 of 36 (69.4%) patients.

**Table 2:** Surgical data

	Total group (*n* = 52)	Hernia width < 10 cm (*n* = 16)	Hernia width ≥ 10 cm (*n* = 36)
Component separation (CS), no (%)			
No CST Open ACS/lateral release Bilateral Unilateral Endo ACS Open TAR Bilateral Unilateral	24 (46.2%) 16 (30.7%) 11 (68.8%) 5 (31.2%) 1 (1.9%) 11 (21.2%) 7 (63.6%) 4 (36.4%)	11 (68.8%) 3 (18.7%) – 3 (100%) – 2 (12.5%) 2 (100%) –	13 (36.1%) 13 (36.1%) 11 (84.6%) 2 (15.4%) 1 (2.8%) 9 (25%) 5 (55.5%) 4 (44.5%)
Mesh repair used, no (%)			
Single mesh Double layer	39 (75%) 13 (25%)	14 (87.5%) 2 (12.5%)	25 (69.4%) 11 (30.6%)
Mesh location, no (%)			
Retro-rectus (sublay) Intra-abdominal (underlay) Inlay Onlay Additional mesh, no (%) Retro-rectus reinforcement Onlay addition Inlay addition	31 (59.6%) 19 (36.5%) 1 (1.9%) 1 (1.9%) *n* = 13 11 (84.6%) 1 (7.7%) 1 (7.7%)	11 (68.8%) 5 (31.2%) – – *n = 2* 2 (100%) – –	20 (55.6%) 14 (38.8%) 1 (2.8%) 1 (2.8%) *n* = 11 9 (81.8%) 1 (9.1%) 1 (9.1%)
Fascial closure, no (%)			
Anterior + posterior fascia closed Anterior fascia closed only Posterior fascia closed only Bridged repair (anterior nor posterior fascia closed)	38 (73.1%) 2 (3.8%) 4 (7.7%) 8 (15.4%)	13 (81.1%) 1 (6.3%) 1 (6.3%) 1 (6.3%)	25 (69.4%) 1 (2.8%) 3 (8.3%) 7 (19.5%)

*CST* component separation technique, *open ACS* anterior component separation (Ramirez), *endo ACS* endoscopic anterior component separation technique, *open TAR* transversus abdominis release

## Discussion

In the present study, we show how intramuscular BTA injections, prior to CAWR, affect the LAW musculature, hernia-, and abdominal dimensions as well as muscle structure. Overall findings show a small but significant decrease of transverse hernia width and LAW muscle thickness, and -muscle mass as well as a significant increase in LAW muscle length. Abdominal width increased, while abdominal depth decreased. Overall, BTA effects are more pronounced in patients with an LoD of at least 20%. In patients with a hernia width of at least 10 cm, BTA decreases hernia width on average, which is not seen in patients with smaller hernia defects. Other dimensions change comparably for larger and smaller hernia defects after BTA. Previous abdominal surgery or hernia repair does not affect the observed BTA effectiveness. BTA, in general, seems less effective in patients with persistent inflammation or contamination.

For most variables individually, we also observed contrasting effects among patients. Consequently, it is best to observe changes in hernia- and abdominal dimensions as a single unit for each individual patient. For example, in a particular patient, an increase instead of a decrease in hernia width may be seen, while LAW muscle length increases significantly or vice versa, resulting in a differential effect on the shape of the abdominal cavity.

Several studies have been published on BTA, but only few have measured effects on LAW muscle length and -thickness and hernia width on CT. Our group have pooled these results in a recent published meta-analysis [14]. In present study, we used the latest computer techniques for assessment of all CT measures. Moreover, we used the correct definitions for these measurements in contrast to definitions used in some of the studies in our meta-analysis. When comparing our present results with previously published data, we observed a smaller effect of BTA for most outcomes. We found a 0.9 cm elongation in LAW muscle length per side in the present study versus as much as 3.2 cm per side in the meta-analysis. LAW muscle thickness has been reported in only one previous study [20] that finds a 1.0 cm decrease on each side versus 0.5 cm per side in the present study. Likewise, we found a 0.4 cm decrease in hernia width versus 3.5 cm in the meta-analysis. No previous studies have reported abdominal cavity width and -depth, making no comparison possible. Furthermore, no previous studies have assessed the mere effect of BTA on LoD without additional use of PPP.

There are several explanations for the differences between our findings and published literature. First, previous studies have included overall larger defects with larger LoD. We did not use a minimum hernia defect size as many of our patients for example had an intestinal fistula or an infected mesh, for which we needed BTA to aid in the complexity of the reconstruction. If we focus in the present series on patients with LoD ≥ 20%, the effects of BTA are more pronounced and more comparable to the findings of the recent meta-analysis; total LAW muscle elongation is 1.7 cm per side with a decrease in LAW muscle thickness of 0.8 cm per side and a decrease of 2.7 cm in hernia width. A second explanation is that we reported medians for our outcome parameters instead of - what has been used frequently - means in aforementioned studies. Medians plus IQRs are less affected by (extreme) outliers. Third, differences in BTA effects across studies can theoretically be caused by differences in BTA techniques applied. We have applied a BTA technique that is commonly used and derived from our meta-analysis of various BTA technical details [14]. Fourth, decrease in hernia width is frequently the focus of BTA effects. Indicated by use of boxplots, however, we showed that there is a considerable proportion of patients with an increase in hernia width instead of a decrease. Patient- and hernia characteristics as well as surgical characteristics were comparable between responding and non-responding patients. Also, for the other abdominal dimensions (abdominal width and -depth and LoD), contrasting results among patients were found, resulting in variable effects on individual abdominal shapes. Since patient-, hernia-, and surgical characteristics could not provide an explanation, possibly, this could be found in the fact that a CT scan does not fully capture the dynamic interplay between hernia-, abdominal cavity-, and abdominal wall dimensions as a unit in each individual patient. Importantly, LAW muscle length and -thickness were the only CT variables that showed a more uniform BTA effect. The heterogeneous results for most CT measures explain why our median observed changes were smaller than expected when looking at effects in an individual patient. Fifth, in the relatively long time between pre-BTA CT and the BTA procedure itself, effects of prehabilitation, mandatory in almost all our patients, play a role.

BTA seems to be less effective in patients with persistent inflammation or contamination due to enterocutaneous or enteroatmospheric fistula, infected mesh, or with persistent intra-abdominal abscess. It is likely that due to the persistent inflammatory or infectious local state, increasing fibrosis formation, the working mechanism of BTA is (partly) diminished in these patients.

No other studies assessing the effect of only BTA have examined its effect on LoD, muscle mass, and -density. Interestingly, no significant overall effect on LoD was observed, but when looking at patients with LoD ≥ 20%, the decrease of LoD becomes larger and significant. This implies that in particular for larger LoD, this is a relevant variable for assessment of BTA effects, in line with the proposal of Sabbagh et al. to use a cut-off value of 20% for LoD as clinically relevant for tension-free fascial closure [37]. In addition, for patients with LoD ≥ 20%, use of BTA may be a prerequisite before hernia repair is planned.

Muscle mass together with muscle density expresses muscle quality. In a rat model, incisional herniation induces LAW muscle shortening and atrophy in which these muscles exhibited significant decreased extensibility and increased stiffness [38]. That incisional hernia induces shortening of the LAW muscles is often visible on CT. When the LAW muscle contraction is (partly) eliminated after BTA, we observed additional atrophy in the LAW muscles expressed by a decreased muscle mass. It is unclear why no significant changes were observed in muscle density. Perhaps, the level of myosteatosis was already reached its maximum because of existing disuse of the muscles due to herniation. The increase in muscle density of the psoas muscles is well explained by offloading of the LAW muscles; all weight bearing of the abdomen is transferred to the (lower) vertebral muscles. Basically, these muscles perform increased exercise which is known to decrease myosteatosis [39].

## Strengths and limitations

The strength of this study lies in its explorative nature. This is the first study that looks at the effects of pretreatment with BTA - without PPP - on different CT measures to quantify the true merits of BTA injections. More knowledge is needed on how these various dimensional results on CT relate to clinical outcomes after hernia repair. Strength is also that all post-BTA CTs were done contrast-enhanced, in a standardized way, at a standardized time interval of 3–6 weeks after BTA and always on the day before surgery.

The limitation of the present study is the variable time interval between initial and post-BTA CT scans. Since the Amsterdam UMC, location AMC is a tertiary referral center for complex abdominal wall reconstructions, initial CTs (pre-BTA CT) had been made at the referring center, and for obvious reasons of patient burden and minimizing radiation burden, no additional CT was done immediately before BTA, which would have been the perfect comparison to the CT 3–6 weeks post-BTA.

## Conclusions

Injection of botulinum toxin induces foremost and most consistently elongation and thinning of the lateral abdominal wall muscles, more than a decrease in hernia width. Concomitantly, the abdomen becomes more oval shaped. An increase in muscle density of the psoas muscles is seen associated with offloading of the LAW muscles. Patients with large LoD have a proportionally larger effect of BTA.

## Supplementary Information

Below is the link to the electronic supplementary material.

Supplementary file1 (PDF 612 KB)
