# Supplementary material for: A computed tomography study investigating the effects of botulinum toxin injections prior to complex abdominal wall reconstruction
Source: Hernia. 2022 Oct 14;27(2):281–91. doi: 10.1007/s10029-022-02692-w (PMC10126041; doi:10.1007/s10029-022-02692-w)
Supplement: Supplementary file 1 — Supplementary file1 (PDF 612 KB) [file 10029_2022_2692_MOESM1_ESM.pdf]

Supplement S1 – Mesh strategy and surgical technique

Mesh strategy

In patients with clean defects a biosynthetic mesh (long-term degradable (Phasix™, CR Bard Inc.) or mid-term degradable (Bio-A®, Gore) was used as single mesh retromuscular (sublay) repair. In large and contaminated / dirty defects, an intra-abdominal (underlay) non-cross-linked biological tissue matrix of porcine dermis (Strattice™ Reconstructive Tissue Matrix, LifeCell) or, later, a polypropylene reinforced biologic tissue matrix of ovine rumen (OviTex® Permanent, TELABio) was used as a leverage with parachuting transfascial sutures to pull close the abdominal cavity and thereby protecting the intra-abdominal viscera. When the anterior fascia could not be closed despite a CST, a dual layer technique was used. This technique comprised an intra-abdominal (underlay) biologic (reinforced) tissue matrix as a leverage and a biosynthetic mesh positioned as retro-rectus (sublay) reinforcement ventral to the biologic tissue matrix creating a dual layer repair.

Surgical techniques

The abdomen was encountered through the previous laparotomy incision, transecting and dissecting but not removing the hernia sac, followed by extensive adhesiolysis. In patients with secondary closed open abdomens usually no real hernia sac is present. Bioburden in case of infected mesh and/or intestinal fistula was reduced by resection of all previously placed mesh and non-viable tissue. Enterocutaneous or enteroatmospheric fistulas were treated by segmental bowel resection, and a hand-sewn double-layer anastomosis was constructed. Up-stream diverting stomas were used infrequently.

Tension-free midline closure of both fascial layers was attempted with mesh reinforcement, preventing a bridged repair whenever possible. When indicated and feasible, component separation techniques were used. If the posterior fascia could not be brought together in the midline, one side of the hernia sac – if present - was used to extend the posterior layer and close the abdominal cavity. The other side of the hernia sac – if present – was used to extent the anterior layer whenever necessary. If the anterior fascia could not be closed primarily without midline tension, or when both fascial layers could not be closed at all, a double layer mesh technique was used (see ‘mesh strategy’).

Suction drains were placed to the surgeon’s discretion (predominantly on the mesh and in the subcutaneous space). Extensive quilting of the subcutaneous space was done. Prophylactic closed incision negative pressure wound therapy (NPWT; Prevena™, KCI, San

Antonio, TX, USA)) for 5-7 days at -125 mmHg has been standard care in our practice since 2014. In the presence of significant loss of skin quality, large full-thickness skin defects and significant loss of domain, skin reconstruction was performed in collaboration with plastic and reconstructive surgeons. Postoperatively all patients were instructed to wear an abdominal binder 24/7 during the first two weeks and subsequent for 3 months when mobilizing. All patients were treated postoperatively according to a complex abdominal wall repair / intestinal failure care pathway.

Supplement S2 - Outcomes stratified, median (IQR)

Variables (cm)	All cases				Loss of domain ≥ 20%				# Abdominal surgery ≥ 3				# Previous hernia repair ≥ 1			
	Difference	%	Sig.	N	Difference	%	Sig.	N	Difference.	%	Sig.	N	Difference	%	Sig.	N
Hernia width	-0.4 (-2.1;0.6)	-2.9	**0.023**	45	-2.7 (-5.5;0.7)	-14.0	0.051	10	-0.4 (-2.4;0.5)	-2.6	**0.032**	33	-0.2 (-2.6;0.6)	-1.3	0.131	21
Abdominal width	0.9 (-0.2;3.3)	3.3	**0.001**	48	2.9 (0.4;3.8)	11.1	**0.022**	10	1.0 (0.1;2.9)	3.7	**0.005**	36	1.7 (0.3;3.3)	6.3	**0.005**	23
Abdominal depth	-0.5 (-1.3;0.5)	-3.5	**0.017**	48	-0.6 (-2.1;0.0)	-5.1	0.139	10	-0.3 (-1.1;0.5)	-2.7	**0.043**	36	-0.3 (-1.0;0.5)	-2.5	0.107	23
LAW muscle length*	0.9 (0.0;2.4)	5.4	**<0.001**		1.7 (0.6;2.6)	10.4	**<0.001**		0.9 (0.0;2.0)	5.4	**<0.001**		1.0 (0.2;2.1)	5.9	**<0.001**	
LAW muscle length R	1.0 (0.2;2.5)	6.0	**<0.001**	48	2.1 (0.5;3.0)	13.5	**0.005**	10	1.0 (0.1;2.4)	6.1	**<0.001**	36	1.0 (0.3;2.5)	6.0	**<0.001**	23
LAW muscle length L	0.7 (-0.3;2.3)	4.1	**<0.001**	51	1.6 (0.5;2.3)	9.5	**0.025**	12	0.6 (-0.5;1.8)	3.6	**0.02**	36	0.8 (-0.5;1.8)	4.7	**0.022**	24
LAW muscle thickness*	-0.5 (-0.8;-0.2)	-25.0	**<0.001**		-0.8 (-1.7;-0.5)	-34.8	**<0.001**		-0.5 (-0.7;-0.2)	-25.0	**<0.001**		-0.5 (-0.8;-0.2)	-23.8	**<0.001**	
LAW muscle thick. R	-0.5 (-0.9;-0.2)	-24.4	**<0.001**	48	-0.8 (-1.8;-0.4)	-31.4	**0.005**	10	-0.5 (-0.8;-0.2)	-24.4	**<0.001**	36	-0.5 (-0.8;-0.2)	-23.8	**<0.001**	23
LAW muscle thick. L	-0.6 (-0.8;-0.2)	-30.0	**<0.001**	50	-0.8 (-1.6;-0.5)	-35.6	**0.003**	12	-0.5 (-0.7;-0.2)	-27.8	**<0.001**	35	-0.5 (-0.8;-0.1)	-26.3	**<0.001**	23
LAW muscle mass (cm^2)*	-3.9 (-6.4;-1.5)	-15.8	**<0.001**		-6.8 (-10.9;-3.5)	-30.3	**0.001**		-4.2 (-6.6;-1.6)	-18.7	**<0.001**		-4.4 (-1.7;-6.6)	-17.1	**<0.001**	
LAW muscle mass R (cm^2)	-4.0 (-6.4;-0.2)	-16.2	**<0.001**	34	-6.8 (-10.4;-4.6)	-29.8	**0.008**	9	-3.2 (-6.5;0.0)	-14.2	**0.001**	24	-4.6 (-6.6;-0.5)	-18.0	**0.005**	16
LAW muscle mass L (cm^2)	-3.7 (-6.5;-2.0)	-15.1	**<0.001**	36	-6.7 (-13.6;0.5)	-31.0	**0.05**	8	-4.4 (-7.2;-2.7)	-19.5	**<0.001**	26	-3.9 (-7.1;-2.6)	-14.8	**0.001**	16
LAW muscle density (HU)*	2.1 (-5.4;7.0)	8.25	0.303		0.98 (-5.4;7.7)	4.6	0.744		2.2 (-6.0;7.4)	8.6	0.463		3.1 (-5.2;8.5)	12.2	0.094	
LAW muscle density R (HU)	1.7 (-6.0;6.0)	6.7	0.726	34	1.8 (-9.6;12.6)	10.5	0.767	9	2.1 (-5.8;6.8)	8.3	0.668	24	1.8 (-5.8;8.9)	7.1	0.469	16
LAW muscle density L (HU)	2.3 (-4.9;7.4	9.0	0.275	36	8.0 (-3.3;12.5)	56.7	0.183	8	0.02 (-5.9;-7.0)	0.1	0.970	26	4.0 (-3.0;7.4)	15.8	0.163	16
Psoas muscle density. (HU)*	4.8 (0.4;9.7)	10.3	**<0.001**	36	5.1 (-3.4;10.8)	11.8	0.208	8	4.8 (0.1;9.8)	10.2	**0.002**	26	5.5 (0.5;11.1)	11.6	**0.005**	16
LoD (%) AR	-0.32 (-2.3;0.7)	-3.1	0.097	50	-3.7 (-12.0;-2.1)	-13.8	**0.012**	12	0.0 (-1.6;1.2)	0.0	0.478	37	-0.4 (-2.3;0.7)	-3.8	0.241	25

* Average per side, *LAW*: lateral abdominal wall, *HU*: Hounsfield units, *LoD*: loss of domain, *AR*: absolute reduction
Difference in N is because of measurements of only intact muscles and/or depending on both pre- and post-BTA CT-scan having contrast.

Supplement S3 - Outcomes grouped by hernia width < 10 cm and ≥ 10 cm and compared, median (IQR)

Variables (cm)	Hernia width < 10 cm			Hernia width ≥ 10cm			*p*-value
	Difference	Sig.	N	Difference	Sig.	N	
Hernia width	0.1 (-0.4;0.5)	0.937	13	-1.0 (-2.4;0.6)	**0.014**	32	0.198
Abdominal width	1.4 (-0.5;3.2)	**0.033**	16	0.9 (-0.3;3.3)	**0.005**	32	0.866
Abdominal depth	-0.4 (-1.4;0.9)	0.352	16	-0.4 (-1.2;0.5)	0.076	32	0.857
LAW muscle length*	0.9 (0.0;3.3)	**0.007**	15	1.0 (0.0;2.3)	**<0.001**	32	0.922
LAW muscle thickness*	-0.5 (-0.9;-0.2)	**<0.001**	15	-0.6 (-0.8;-0.2)	**<0.001**	31	0.695
LAW muscle mass (cm^2)*	-3.0 (-5.6;0.1)	**0.005**	12	-4.4 (-7.2;-2.1)	**<0.001**	21	0.095
LAW muscle density (HU)*	4.7 (-3.4;7.4)	0.260	10	0.2 (-6.0;7.1)	0.553	21	0.472
Psoas muscle density. (HU)*	6.9 (-1.3;10.5)	**0.034**	12	4.1 (-0.2;9.4)	**0.006**	21	0.546
LoD (%) AR	0.0 (-1.2;0.2)	0.388	15	-0.9 (-2.7;1.2)	0.140	31	0.743

* Average per side, *LAW*: lateral abdominal wall, *HU*: Hounsfield units, *LoD*: loss of domain, *AR*: absolute reduction
Difference in N is because of measurements of only intact muscles and/or depending on both pre- and post-BTA CT-scan having contrast.

Supplement S4 - Patient- and hernia characteristics and surgical characteristics grouped according to hernia width decrease or no decrease after BTA

	Decrease hernia width after BTA (n=25)	No decrease hernia width after BTA (n=20)	*p*-value
BMI >30 (kg/m²), no (%)	8 (32%)	7 (35%)	0.834
Complicating hernia characteristics(ǂ), no (%)			0.441
0	2 (8%)	3 (15%)	
1-2	12 (48%)	10 (50%)	
≥ 3	11 (44%)	7 (35%)	
Loss of domain ≥ 20 (%), no (%)	6 (24%)	4 (20%)	0.751
Previous abdominal surgery, no (%)			0.161
1-2	4 (16%)	5 (25%)	
3-4	8 (32%)	9 (45%)	
≥ 5	13 (52%)	6 (30%)	
Previous hernia repair, no (%)			0.901
0	13 (52%)	10 (50%)	
1	6 (25%)	5 (25%)	
≥ 2	6 (26.9%)	5 (25%)	
Component separation (CS), no (%)			0.669
no CST	11 (44%)	8 (40%)	
open ACS	9 (36%)	7 (35%)	
endo ACS	1 (4%)	-	
open TAR	4 (16%)	5 (25%)	
Fascial closure, no (%)			0.345
anterior + posterior fascia closed	20 (80%)	13 (65%)	
anterior fascia closed only	1 (4%)	-	
posterior fascia closed only	-	3 (15%)	
bridged repair (anterior nor posterior fascia closed)	4 (16%)	4 (20%)	

(ǂ) Including: presence of a stoma, intestinal fistula, infected mesh, transverse defect width ≥10cm, loss of domain >20%, previous hernia repair).
CST: component separation technique, *open ACS*: anterior component separation (Ramirez), *endo ACS*: endoscopic anterior component separation technique, *open TAR*: transversus abdominis release. *BMI*: body mass index.

Supplement 5 - Outcomes grouped by persistent inflammation/contamination during BTA, median (IQR)

Variables (cm)	Without inflammation or contamination			With inflammation and contamination			*p*-value
	Difference	Sig.	N	Difference	Sig.	N	
Hernia width	0.0 (-2.5;0.7)	0.179	29	-0.5 (-2.0;0.4)	0.078	16	0.749
Abdominal width	1.5 (-0.2;3.4)	**0.001**	29	0.7 (-0.9;1.5)	0.501	16	**0.035**
Abdominal depth	-0.2 (-1.4;0.8)	0.234	29	-0.6 (-1.2;-0.2)	**0.003**	16	0.29
LAW muscle length*	1.0 (0.3;2.7)	**<0.001**	32	0.6 (-0.3;2.2)	**0.047**	15	0.191
LAW muscle thickness*	-0.6 (-0.9;-0.2)	**<0.001**	30	-0.4 (-0.7;-0.2)	**<0.001**	15	0.111
LAW muscle mass (cm^2)*	-3.7 (-6.4;-2.0)	**<0.001**	19	-3.6 (-6.3;-0.1)	**<0.001**	13	0.838
LAW muscle density (HU)*	2.3 (-4.9;6.9)	0.315	21	1.9 (-5.7;7.3)	0.758	11	0.762
Psoas muscle density. (HU)*	6.2 (0.6;10.7)	**0.001**	22	3.6 (-3.0;7.7)	0.221	14	0.074
LoD (%) AR	-0.4 (-2.8;1.0)	0.108	32	0.0 (-2.3;0.7)	0.427	18	0.585

^ Inflammation/contamination includes enterocutaneous or –atmospheric fistula (ECF/EAF), infected meh, intra-abdominal abscess.
* Average per side, *LAW*: lateral abdominal wall, *HU*: Hounsfield units, *LoD*: loss of domain, *AR*: absolute reduction
Difference in N is because of measurements of only intact muscles and/or depending on both pre- and post-BTA CT-scan having contrast.
